# Safety range of free valproic acid serum concentration in adult patients

**DOI:** 10.1371/journal.pone.0238201

**Published:** 2020-09-02

**Authors:** Yu-Ju Tseng, Shih-Ying Huang, Chih-Hsuan Kuo, Chen-Yu Wang, Kuo-Chuan Wang, Chien-Chih Wu

**Affiliations:** 1 Department of Pharmacy, National Taiwan University Hospital, College of Medicine, National Taiwan University, Taipei, Taiwan; 2 Department of Laboratory Medicine, National Taiwan University Hospital, College of Medicine, National Taiwan University, Taipei, Taiwan; 3 School of Pharmacy, College of Medicine, National Taiwan University, Taipei, Taiwan; 4 Graduate Institute of Clinical Pharmacy, College of Medicine, National Taiwan University, Taipei, Taiwan; 5 Department of Pharmacy, National Taiwan University Hospital Yun-Lin Branch, College of Medicine, National Taiwan University, Douliu City, Yun-Lin County, Taiwan; 6 Division of Neurosurgery, Department of Surgery, National Taiwan University Hospital, College of Medicine, National Taiwan University, Taipei, Taiwan; University of Catanzaro, ITALY

## Abstract

**Background:**

Therapeutic drug monitoring (TDM) is recommended during valproic acid (VPA) use, and total serum concentration has been widely adopted. However, the free form of VPA is responsible for its pharmacologic and toxic effects, and the total and free concentrations are highly discordant because of VPA’s highly protein bound and saturable binding characteristics. Therefore, free VPA monitoring is increasingly advocated. Nevertheless, the correlation between free VPA concentration and associated adverse effects remains unknown.

**Objective:**

To determine the optimal safety range of free VPA concentration in adult patients.

**Materials and methods:**

This prospective cohort study enrolled adult patients undergoing VPA therapy with TDM. Patient characteristics, VPA use, and adverse effects (thrombocytopenia, hyperammonemia, and hepatotoxicity) were recorded. A multivariate logistic regression model was applied to identify the predictors of adverse effects, and the receiver operating characteristic curve was applied to locate the cutoff point of free VPA concentration.

**Results:**

A total of 98 free serum concentrations from 51 patients were included for final analysis. In total, 31 (31.6%), 27 (27.6%), and 4 (4.1%) episodes of hyperammonemia, thrombocytopenia, and hepatotoxicity were observed, respectively. Free VPA concentration was a predicting factor for thrombocytopenia but not for hyperammonemia. A free VPA concentration of >14.67 mcg/mL had the greatest discriminating power (area under the curve = 0.77) for the occurrence of thrombocytopenia.

**Conclusions:**

A free VPA serum concentration of 14.67 mcg/mL had the optimal discriminating power for the occurrence of thrombocytopenia. Ammonemia should be monitored even if free VPA concentration is within the safety range.

## Introduction

Valproic acid (VPA) has been used extensively as migraine prophylaxis and in the treatment of seizure, bipolar disorder, and other psychiatric or neurological conditions. Its mechanism is complex, and it is involved in potentiating the γ-aminobutyric acid effect and attenuating neuronal response to *N*-methyl-D-aspartate, which leads to inhibited neuronal excitation [1, 2]. VPA also modulates neurotransmitters such as dopamine, serotonin, and voltage-gated ion channels such as sodium, calcium, and potassium channels that stabilize neuronal membranes [2]. Concerning pharmacokinetics, VPA typically has high bioavailability (90%), and albumin binding is commonly reported at 90%, but it may be much less than this in specific situations, including in low albumin states, with elevated blood urea nitrogen (BUN), and with medications that compete for binding such as lipid emulsion agents, aspirin, and phenytoin [3–6]. VPA is extensively metabolized by the liver through glucuronidation (50%), mitochondrial β-oxidation (40%), and other pathways. Less than 3% of the administered dose of VPA is excreted unchanged in the urine [1].

Therapeutic drug monitoring (TDM) is recommended to ensure the efficacy and safety of VPA. Total VPA serum concentration, when measured clinically, has a recommended therapeutic range of 50–100 mcg/mL [7]. However, VPA is highly bound to albumin, and the binding capacity becomes saturated when the serum concentration increases, resulting in higher amounts of free (unbound) VPA [5]. Free VPA is responsible for the drug’s pharmacologic effects but also for its toxicity. At a normal albumin concentration and low total serum concentration (20–60 mcg/mL), the unbound fraction of VPA is approximately 10% of the total serum concentration [8]. The unbound fraction might exceed 15% when total serum concentration is higher than 100 mcg/mL [8]. In addition to high VPA serum concentration, hypoalbuminemia is another critical factor that increases the unbound fraction of VPA, potentially increasing toxicity, particularly for inpatients [4]. Haley et al. [9] demonstrated that the unbound fraction of VPA was significantly higher for inpatients who typically have lower albumin levels than outpatients. Other circumstances may increase the unbound fraction of VPA, including the presence of endogenous substances that compete with VPA for albumin (e.g., bilirubin, fatty acids, and urea nitrogen) and concomitant medications (e.g., aspirin, nonsteroidal anti-inflammatory drugs, phenytoin, warfarin, propofol, clevidipine, and lipid infusions) [3]. The unbound fraction could be greater than 20% in patients in critical care [10, 11].

All of these factors can increase the unbound fraction of VPA, resulting in a higher risk of poisoning, and the TDM of total VPA serum concentration may be inadequate because of the highly discordant free VPA serum concentration [12, 13]. Free VPA concentration is reportedly a more accurate predictor of adverse neurological symptoms than total VPA concentration [4]. Because of the difficulty of predicting free VPA serum concentration and the inaccuracy of albumin-based normalizing formula for total serum concentration, researchers have advocated for more direct monitoring of free VPA serum concentration [7, 10, 11]. Although various therapeutic ranges of free VPA serum concentration, such as 5–15 or 7–23 mcg/mL, have been proposed in the literature, the optimal range remains undetermined [14, 15]. This study investigated the relationship between free VPA serum concentration and adverse effects to determine the optimal clinical safety range.

## Materials and methods

This prospective cohort study was conducted from April 1, 2018 to March 31, 2019 at National Taiwan University Hospital (NTUH), which is a 2600-bed tertiary referral center in northern Taiwan. This study was approved by the Institutional Review Board (201801129RIND) of NTUH, and informed consent was acquired from all participants or a legal representative. Adult patients aged over 20 years who were receiving VPA therapy and whose total VPA serum concentration was being monitored were included in this study. The residual sample was used for measurement of free VPA serum concentration. The sampling time for VPA serum concentration was within 1 hour prior to receiving the next dose and at least 3 days after VPA initiation or dosage adjustment. If the free VPA serum concentration was undetectable or sampling time was earlier than 1 hour prior to the next dose, the data were excluded. Each VPA TDM was regarded as a new episode; thus, patients could be enrolled repeatedly. All enrolments were included in the safety analysis.

The following data were collected during each VPA TDM: participants’ age, sex, height, body weight, ward type, comorbidities, concomitant medications with the potential to interact with VPA, shock status (defined by vasopressor use), and VPA use (the indications, dosage, route of administration, and total and free trough serum concentration [within 1 hour before the next dose]). The following lab data were also recorded for each VPA TDM: albumin, BUN, serum creatinine, ammonia (NH_3_), total bilirubin, alanine aminotransferase (ALT), and platelets. Because we enrolled patients with neurologic problems, evaluating and attributing subjective side effects, such as nausea, vomiting, and drowsiness, to VPA use rather than to disease progression was challenging. Therefore, thrombocytopenia, hyperammonemia, and hepatotoxicity were used to evaluate the safety range of free VPA serum concentration. Adverse effects were evaluated by a clinical pharmacist who employed the Naranjo score, and only a score of ≧ 5 points was considered to be associated with VPA [16]. Hyperammonemia, hepatotoxicity, and thrombocytopenia were defined as NH_3_ > 60 μmol/L, ALT > 3 times upper normal limit (UNL) (or > 2 times the pretreatment value if that value > 3 times UNL), and platelet count < 140,000 cells/mm3 (or a decrease of more than 50% relative to the pretreatment value) after VPA use, respectively [10].

The total VPA serum concentration was measured using an immunoassay analyzer (ABBOT ARCHITECT i-2000SR, Abbott Park, Illinois, USA), which has a detection range of 2.00–150 mcg/mL. For free VPA serum concentration, a blood sample was first processed with a filter device (Amicon Ultra-0.5 10K device, Merck Millipore, Ireland) to remove the albumin, and then the concentration was measured using the aforementioned immunoassay analyzer.

Data are described as median with range or numbers with percentages. The Mann–Whitney U test was used for continuous data, and either chi-square or Fisher’s exact test was used for categorical data. A p-value ≤ 0.05 was considered statistically significant. A multivariate logistic regression model was constructed to identify the predictors of adverse effects using variables for which the p-value was less than 0.1 in the univariate analysis. The receiver operating characteristic (ROC) curve was applied to determine the cutoff point of free VPA concentration for safety, which was represented by the maximum Youden index. All statistical analyses were performed using SAS software (version 9.3; SAS Institute Inc., Cary, NC, USA).

## Results

A total of 98 serum concentration data points from 51 participants were included for final analysis. Of the 98 TDM episodes, 36 involved women (37%) and 95 involved inpatients (97%), of whom 48% were admitted in intensive care units (Table 1). The age and baseline platelet count were 70 yrs (25–96) and 211 K/μL (66–466), respectively. VPA was mainly used for focal seizure (42%), generalized tonic-clonic seizure (33%), status epilepticus (4%), and postherpetic neuralgia (1%). VPA was also used for post-hemorrhage stroke (10%) and as a post-neurosurgery seizure prophylaxis (10%). The oral form was used for 60% of the episodes, and the median daily dose was 22.18 (16.80–30.64) mg/kg. The total and free VPA serum concentrations were 46.55 (11.2–121.45) and 9.41 (2.32–57.67) mcg/mL, respectively. The free fraction was 19.53% (9.47%–54.46%), and hypoalbuminemia (<3.5 g/dL) occurred in 77% of episodes.

**Table 1:** Demographic and clinical characteristics for TDM episodes.

Characteristics	N = 98
Age (yr)	70 (25–96)
Female sex no. (%)	36 (37)
Body weight (kg)	60.1 (35.8–91)
Comorbidities	
Liver disease, no.(%)	9 (9)
Chronic kidney disease, no.(%)	16 (16)
VPA indication	
Seizure prophylaxis no. (%)	20 (20)
Post-hemorrhage stroke, no.	10 (10)
Post-neurosurgery, no.	10 (10)
Seizure treatment no. (%)	78 (80)
Focal seizure, no.	42 (42)
Generalized tonic-clonic seizure, no.	32 (33)
Status epileptics, no.	3 (4)
Post-herpetic neuralgia	1 (1)
Outpatient no. (%)	3 (3)
Inpatient, no. (%)	95 (97)
ICU, no. (%)	47 (48)
General ward, no. (%)	48 (49)
Dosage form	
Oral Solution	47 (48)
Chrono tablet	12 (12)
Intravenous	39 (40)
Daily dose (mg/kg/day)	22.18 (16.80–30.64)
Shock, no. (%)	12 (12)
Albumin(g/dL)	3.05 (2.0–4.4)
≧3.5, no. (%)	16 (16)
3.5 > albumin≧2.5, no. (%)	75 (77)
< 2.5, no. (%)	7 (7)
Total bilirubin (mg/dL)	0.43 (0.15–1.68)
BUN (mg/dL)	16.9 (4.5–102.6)
PLT (K/μL)	211 (66–466)
NH_3_ (μmol/L)	36 (24–73)
ALT (U/L)	15 (4–57)
Concomitant medication	
Acetaminophen, no. (%)	30 (31)
Aspirin, no. (%)	11 (11)
NSAIDs, no. (%)	3 (3)
Phenytoin, no. (%)	4 (4)
Warfarin, no. (%)	2 (2)
Total VPA concentration (mcg/mL)	46.55 (11.2–121.45)
Free VPA concentration (mcg/mL)	9.41 (2.32–57.67)
< 5, no. (%)	14 (14)
5–15, no. (%)	65 (66)
>15, no. (%)	19 (20)
Free fraction (%)	19.53 (9.47–54.46)

Data presented as median (range) or numbers (percentages).

ALT, alanine aminotransferase; ICU, intensive care unit; BUN, blood urea nitrogen; NH_3_, ammonia; NSAID, nonsteroidal anti-inflammatory drugs; PLT, platelet; TDM, therapeutic drug monitoring; VPA, valproic acid.

**Fig 1:**
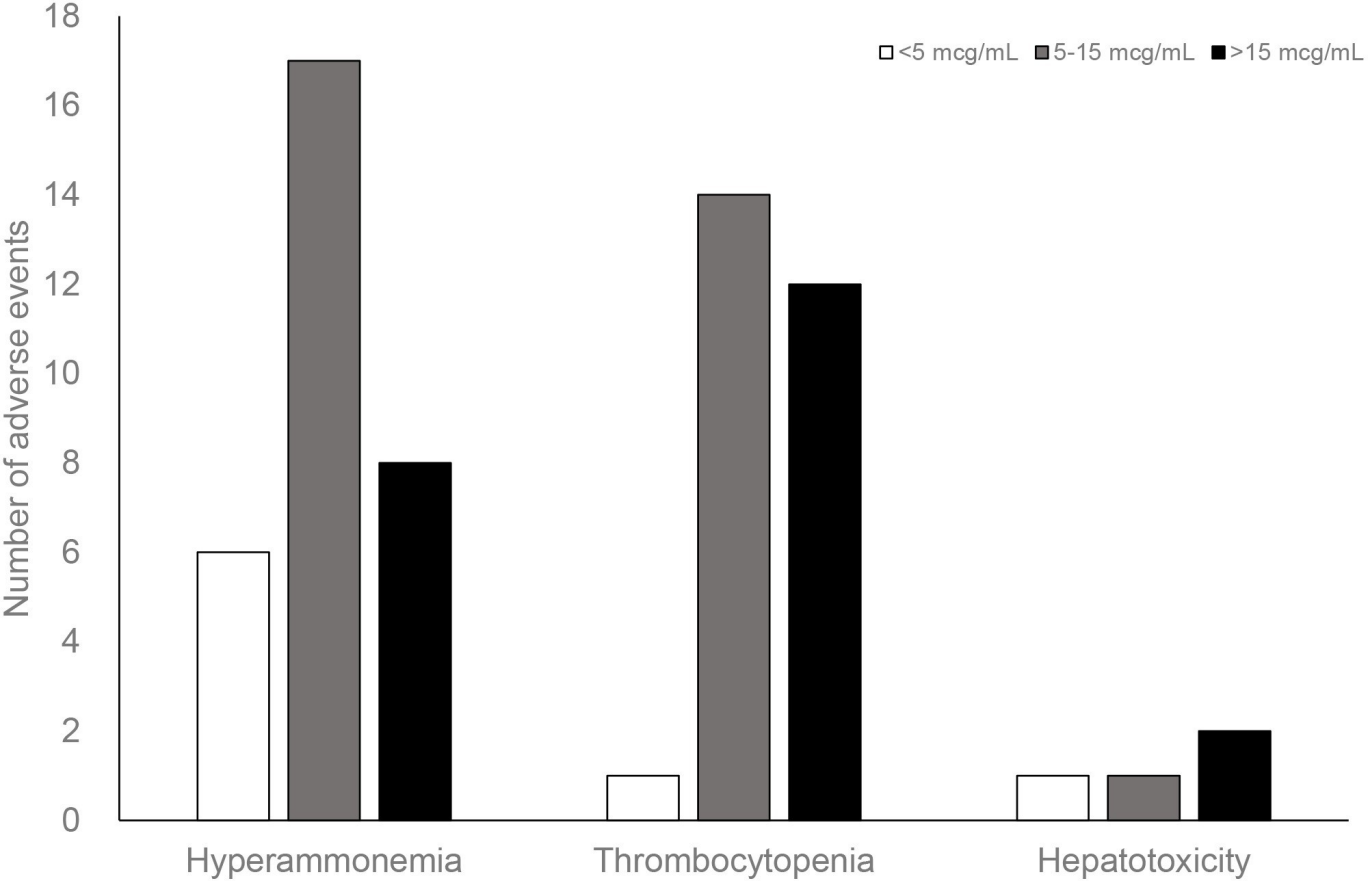
Distribution of free serum concentration to valproic acid–associated adverse effects.

**Fig 2:**
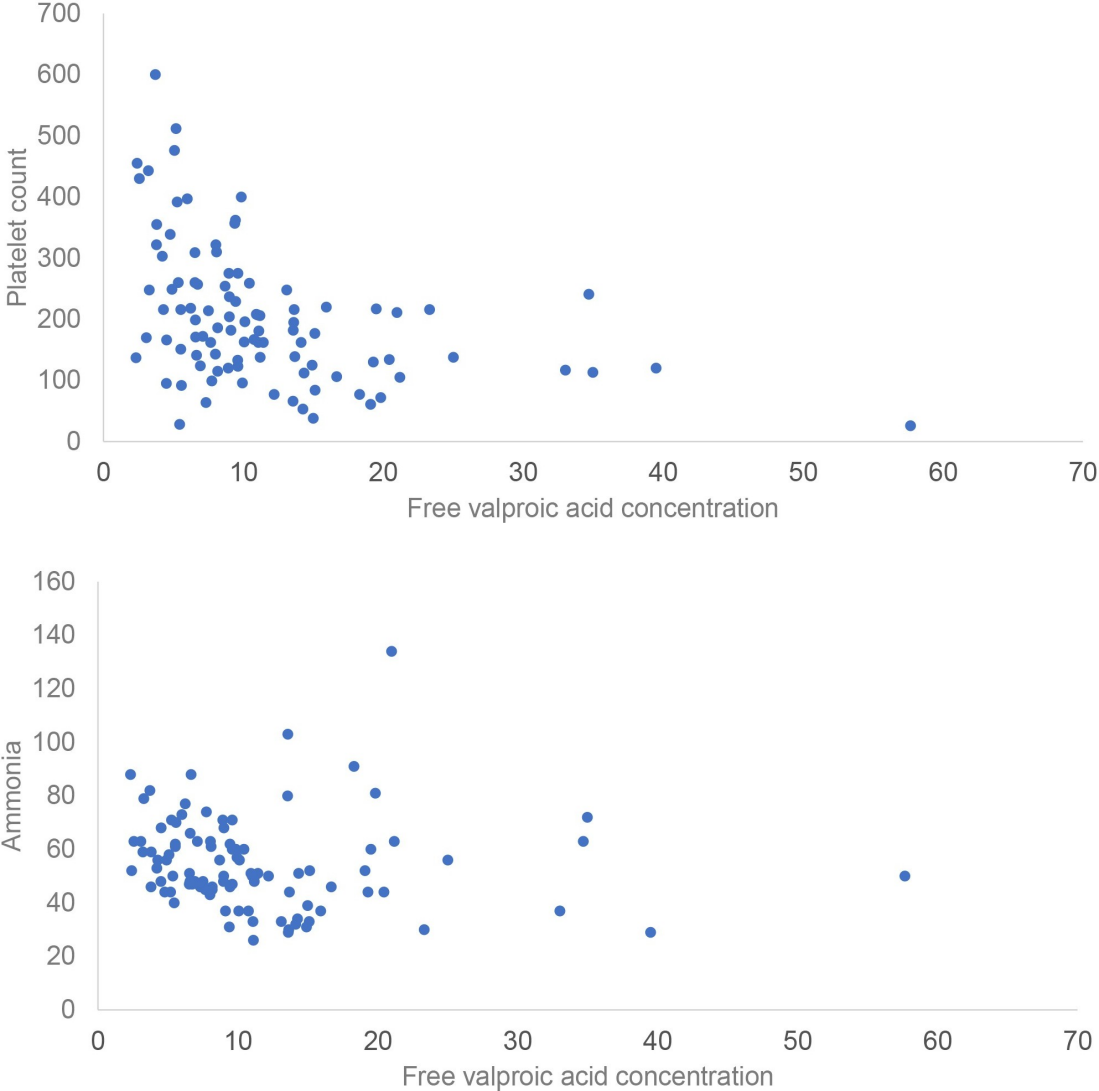
Scatterplot of adverse effect and free valproic acid serum concentration. (a) Platelet. (b) Ammonia.

**Fig 3:**
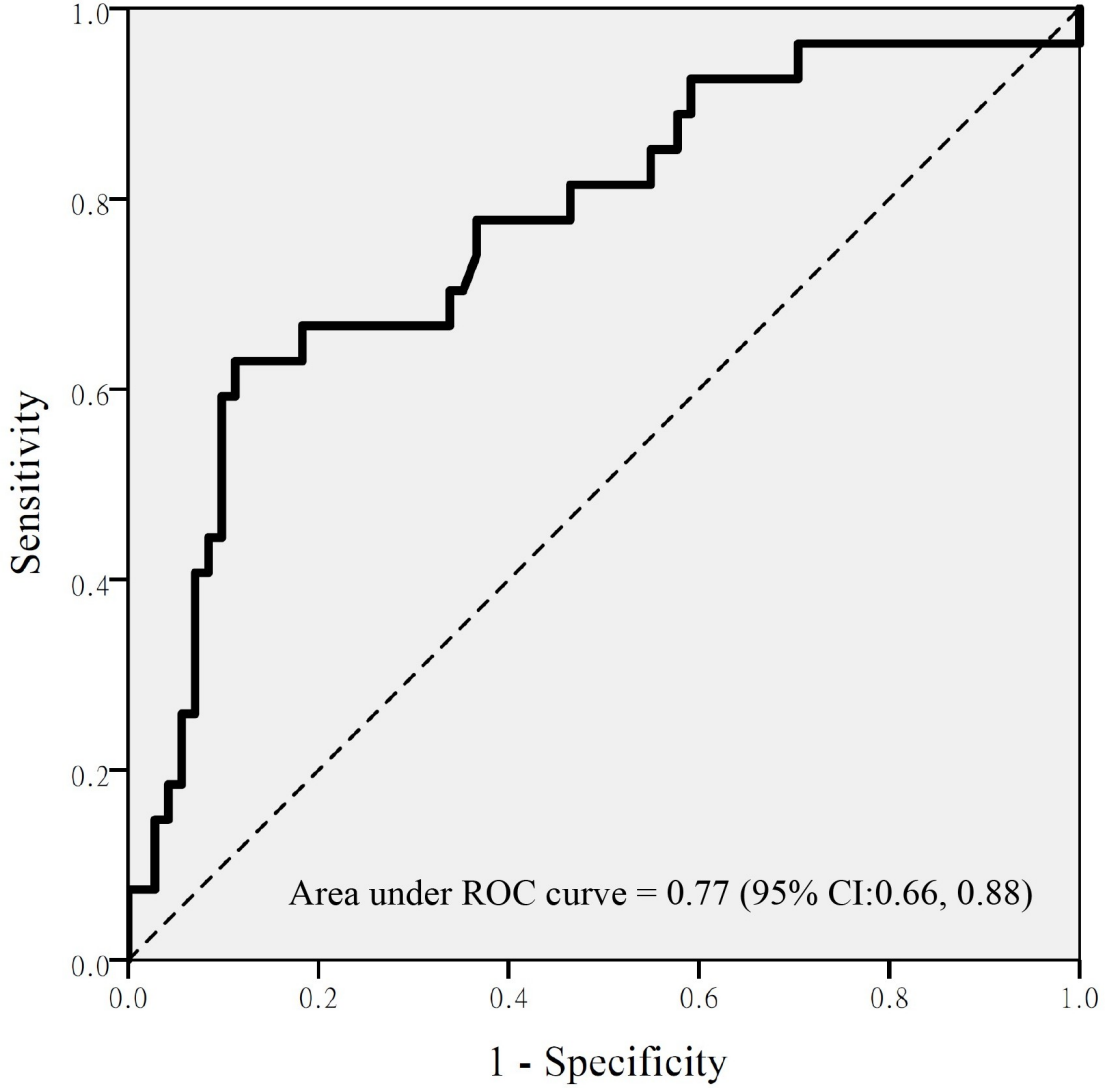
Receiver operating characteristic curve of free valproic acid concentration at the cutoff value of 14.67 for the prediction of thrombocytopenia. CI: confidence interval.

In total, 31, 27, and 4 episodes of hyperammonemia, thrombocytopenia, and hepatotoxicity (Fig 1) were observed. The Pearson correlation coefficient was 0.42 (p < 0.001) between platelet count and free VPA concentration and 0.1 (p = 0.33) between ammonia and free VPA concentration (Fig 2). Patients with thrombocytopenia episodes were older [72(29–89) vs. 67(25–96), p = 0.02] and had lower baseline platelet count [162(93–331) vs. 219(66–446), p < 0.001] than those without. In addition, free and total VPA concentrations were significantly higher in the thrombocytopenia group than those in the non-thrombocytopenia group. However, this difference was not found for hyperammonemia and hepatotoxicity (Table 2). Age, free VPA serum concentration, total bilirubin, and baseline platelet count were predicting factors for thrombocytopenia (Table 3); however, no significant risk factors were found for hyperammonemia (Table 4). Logistic regression was not performed for hepatotoxicity because of the rarity of this adverse effect in the participants. The ROC curve indicated that a free VPA concentration > 14.67 mcg/mL had the greatest discriminating power for the occurrence of thrombocytopenia. The area under the ROC curve was 0.77 (95% confidence interval: 0.66–0.88, p < 0.001) (Fig 3). The sensitivity and specificity of this cutoff value were 48.1% and 88.7%, respectively.

**Table 2:** Association of adverse effects and free or total valproic acid concentration.

Adverse effect	Free VPA (mcg/mL)	P-value	Total VPA (mcg/mL)	P-value
Thrombocytopenia	14.9 (2.3–57.7)	<0.001	59.4 (11.2–121.5)	<0.0001
Non-thrombocytopenia	8.2 (2.4–35.0)		43.0 (19.3–106.4)	
Hyperammonemia	8.9 (2.4–57.7)	0.45	46.0 (20.5–121.5)	0.95
Non-hyperammonemia	9.6 (2.3–39.5)		46.7 (11.2–104.4)	
Hepatotoxicity	19.1 (2.4–34.7)	0.88	68.6 (20.5–106.4)	0.74
Non-hepatotoxicity	9.41 (2.3–57.7)		46.5 (11.2–121.5)	

Data presented as median (range); VPA, valproic acid.

**Table 3:** Factors associated with the occurrence of thrombocytopenia.

	Univariate		Multivariate*	
	OR (95%CI)	P-value	OR (95%CI)	P-value
Male sex	1.55 (0.60–4.01)	0.37		
Age	1.05 (1.02–1.09)	0.003	1.05 (1.01–1.09)	0.03
Free VPA concentration	1.12 (1.05–1.21)	0.001	1.10 (1.01–1.19)	0.02
Albumin	0.64 (0.23–1.79)	0.39		
BUN	1.01 (0.99–1.03)	0.31		
Total bilirubin	6.12 (1.25–30.06)	0.03	15.55 (1.36–177.25)	0.03
Acetaminophen	0.42 (1.14–1.24)	0.12		
High protein binding medication^#^	0.85 (0.28–2.62)	0.78		
ICU	1.22 (0.50–2.96)	0.67		
Shock	3.91 (0.90–10.63)	0.07	2.79 (0.39–20.02)	0.31
Baseline platelet count	0.98 (0.98–0.99)	0.02	0.99 (0.98–1.00)	0.01
Liver disease	2.30 (0.57–9.29)	0.24		
Kidney disease	0.56 (0.15–2.14)	0.39		

CI, confidence interval; ICU, intensive care unit; OR, odds ratio; VPA, valproic acid; BUN, blood urea nitrogen.

#: protein binding > 80%.

*:Age, free VPA concentration, total bilirubin, shock and baseline platelet count were variables for multivariate analysis. The Nagelkerke's R^2^ = 0.46.

**Table 4:** Factors associated with the occurrence of hyperammonemia.

	Univariate logistic regression	
	OR (95% CI)	P-value
Male sex	1.08 (0.45–2.63)	0.86
Age	1.01 (0.98–1.03)	0.67
Free VPA concentration	1.02 (0.97–1.07)	0.53
Albumin	0.82 (0.31–2.15)	0.69
BUN	0.98 (0.96–1.00)	0.06
Total bilirubin	1.34 (0.30–6.01)	0.70
Acetaminophen	1.39 (0.56–3.44)	0.48
High protein binding medication^#^	0.91 (0.31–2.64)	0.86
ICU	0.98 (0.42–2.29)	0.95
Shock	1.65 (0.48–5.58)	0.43
Baseline ammonia	0.98 (0.94–1.02)	0.38
Liver disease	0.59 (0.12–3.03)	0.53
Kidney disease	0.68 (0.20–2.30)	0.54

CI, confidence interval; ICU, intensive care unit; OR, odds ratio; VPA, valproic acid; BUN, blood urea nitrogen.

#: protein binding > 80%.

## Discussion

VPA is widely administered for seizure treatment, and both its efficacy and adverse effects are highly related to its concentration; therefore, TDM is recommended during its clinical use. Although several studies have demonstrated that free serum concentration has a stronger association with adverse effects than does total serum concentration, total serum concentration is adopted more widely for technical and economic reasons [4, 17]. However, free and total VPA serum concentrations are highly discordant because of the unpredictable free fraction, particularly in case of higher total VPA serum concentration (>60 mcg/mL) and hypoalbuminemia [4, 8, 9]. Riker et al. [10] analyzed VPA use in 15 patients in an intensive care unit and reported a considerable variation in free fraction, ranging from 15% to 89%. Although a normalized formula with albumin is favored by some because of its stronger correlation between total and free VPA serum concentration, its accuracy remains questionable. Drisaldi et al. [11] investigated 174 patients and found that using albumin for the correction of total VPA serum concentration resulted in poor concordance (56.9%) with measured free concentration. Because of these limitations of total serum concentration, free concentration monitoring is advocated. However, no consensus has been reached regarding the safe range of free VPA serum concentration.

To the authors’ knowledge, this is the first study to explore the optimal safety range of free VPA serum concentration in adult patients according to adverse effects with the use of objective measurements. Dore et al. [4] reported that free VPA concentration was associated with adverse neurological symptoms, whereas total VPA concentration was not. However, confounding factors such as neurological comorbidities or concurrent medications that might also lead to adverse neurological effects were not adjusted, which may have affected the robustness of the results. Free VPA serum concentration is a predicting factor for thrombocytopenia but not for hyperammonemia. The mechanism of VPA-induced thrombocytopenia remains unclear. The incidence rate of thrombocytopenia was 28% in this study, which is compatible with that described in related reports (0%–28%) [18–22]. Nasreddine et al. [21] reported an incidence rate of 17.7% and identified female sex and low baseline platelet count as risk factors. Furthermore, they proposed that higher total VPA serum concentration (>100 mcg/mL for males and >130 mcg/mL for females) raised the risk of thrombocytopenia. Age, female sex, and high doses of VPA are also reportedly risk factors for thrombocytopenia [22]. Therefore, thrombocytopenia is thought to be a dose-dependent adverse effect of VPA, which is compatible with our result that higher free VPA serum concentration is a risk factor for thrombocytopenia, and a free VPA serum concentration of 14.67 mcg/mL had the optimal discriminating power for the occurrence of thrombocytopenia.

The frequency of hyperammonemia in adult patients is highly variable, ranging from 27.8% to 55.3% [23–25]. The incidence of hyperammonemia in the current study was 31.6%, which is compatible with rates reported in other studies. Although the exact mechanism of VPA-induced hyperammonemia remains unclear, it might be related to the direct inhibition of carbamoyl phosphate synthetase (CPS) I by VPA metabolites such as valpronyl-CoA and propionate, or the indirect effect on CPS I resulting from the downregulation of N-acetylglutamate by valpronyl-CoA and propionate. Yamamoto et al. [25] revealed that total daily dose is a risk factor for hyperammonemia; however, they did not include total serum concentration in the regression model. Although Tseng et al. [24] reported a positive correlation between total VPA serum concentration and ammonia level, the correlation was relatively weak (r = 0.21). Itoh et al. [17] found that free VPA concentration was more strongly correlated with hyperammonemia than total concentration was, and higher free VPA concentration (>8.65 mcg/mL) increased the risk of hyperammonemia; however, no confounding factors were adjusted. In this study, we explored the relationship between free VPA serum concentration and hyperammonemia; however, we did not observe a significant correlation. This may be attributed to the different status of seizure control. Uncontrolled seizure may also cause hyperammonemia, but seizure status was not adjusted in a previous and the current study [26]. Further large studies are required to explore the correlation between free VPA serum concentration and hyperammonemia.

This study has some limitations. First, we only enrolled adult patients and included only three outpatients; therefore, the results may not be generalizable to the pediatric population and outpatients. Second, only patients undergoing TDM were enrolled in our study; therefore, selection bias may have existed. Third, multiple concentrations for each patient were used for analysis, which may have affected the robustness of our results. We used the last value for each patient for sensitivity analysis, which revealed that free VPA serum concentration and baseline platelet count were still significant predictors for the occurrence of thrombocytopenia, further strengthening our result. Lastly, we did not evaluate the association between free VPA serum concentration and efficacy. Free VPA concentrations lower than the safety range may be associated with reduced seizure control. Further research should evaluate the association between free VPA serum concentration and efficacy to determine the optimal therapeutic range.

## Conclusions

Free VPA serum concentration seems to be a risk factor for thrombocytopenia but not for hyperammonemia. The value of 14.67 mcg/mL exhibited the optimal discriminating power for the occurrence of thrombocytopenia. Ammonia should be closely monitored even if free VPA serum concentration is within the safety range.

## Supporting information

S1 File Data set for analysis. (XLS)
